# Low Pathogenic Strain of Infectious Pancreatic Necrosis Virus (IPNV) Associated with Recent Outbreaks in Iranian Trout Farms

**DOI:** 10.3390/pathogens9100782

**Published:** 2020-09-24

**Authors:** Sohrab Ahmadivand, Manfred Weidmann, Mansour El-Matbouli, Hooman Rahmati-Holasoo

**Affiliations:** 1 Department of Aquatic Animal Health, Faculty of Veterinary Medicine, University of Tehran, Tehran P.O. Box 14155-6453, Iran; rahmatih@ut.ac.ir; 2 Institute of Aquaculture, University of Stirling, Stirling FK9 4LA, Scotland, UK; m.w.weidmann@stir.ac.uk; 3 Clinical Division of Fish Medicine, University of Veterinary Medicine, 1210 Vienna, Austria; Mansour.El-Matbouli@vetmeduni.ac.at

**Keywords:** VP2, Sp serotype, rainbow trout, pathology, virulence

## Abstract

Infectious pancreatic necrosis (IPN), first described as acute viral catarrhal enteritis, is a highly contagious disease with variable pathogenicity that has been linked to genetic variation in the viral VP2 gene encoding the capsid protein. In this study, the IPN virus (IPNV) is isolated from the moribund fish from five of fourteen Iranian trout farms from 2015 to 2017. The affected fish showed mortality rates ranging from 20% to 60%, with the main clinical signs of exophthalmia, darkened skin, and mild abdominal distension, as well as yellow mucoid fluid in the intestine. Histopathological examination of intestinal sections confirmed acute catarrhal enteritis in all samples. RT-PCR assay of the kidney tissue and cell culture (CHSE-214) samples consistently confirmed the presence of the virus. The phylogenetic analysis of the partial VP2 sequence revealed that the detected isolates belong to genogroup 5, and are closely related to the Sp serotype strains of European origin. Characterization of VP2 of all isolates revealed the P_217_T_221_ motif that previously was associated with avirulence or low virulence, while all IPNV-positive fish in this study were clinically affected with moderate mortality. The IPNV isolates from Iran are associated with two lineages that appear to have originated from Europe, possibly via imported eggs.

## 1. Introduction

Aquaculture, owing to its rapid expansion and interconnected international production operations (including extensive trade of eyed eggs and animals at different life stages), creates conditions in which viruses and other pathogens can spread [1].

Iran is one of the world-leading producers of freshwater rainbow trout with an annual production above 100k tons, and a respective annual demand for 300–400 million eyed eggs, of which 70% are imported from European countries. In recent years, the industry faced significant losses, due to viral diseases, such as rhabdoviruses and IPNV, which was first reported in the late 2000s, and since has become endemic [2,3,4,5].

Infectious pancreatic necrosis virus (IPNV) is the causative agent of the highly contagious and acute catarrhal enteritis named infectious pancreatic necrosis disease (IPN), mainly affecting farmed salmonid fish worldwide [6,7].

IPNV is a non-enveloped virus with a bi-segmented (A and B) dsRNA genome (~5 kbp) and belongs to the *Aquabirnavirus* genus within the *Birnaviridae* family [8]. Segment A encodes two viral structural proteins VP2 and VP3, the protease VP4, and a nonstructural protein, VP5 with indefinite function [7,9,10]. Segment B encodes VP1, a virion-associated RNA-dependent RNA polymerase [11].

IPNV isolates were classified into two serogroups comprising 10 serotypes (A1–A9 and B) among which, all except for Tellina virus-1 within serogroup B are pathogenic to fish [12]. Moreover, the sequence analysis of VP2 revealed the existence of seven genogroups according to serotypes and geographical origins [13,14].

The symptoms of IPN include spiral swimming, skin darkening, exophthalmia, abdominal distension, and often pale gills [15]. The disease usually occurs at temperatures between 10 °C to 15 °C, with 5–90% mortality depending on the strain and quantity of the virus, the host species, and age, as well as the environmental condition [7].

IPNV mainly causes high mortality in fry in freshwater and post-smolts shortly after the transfer to seawater, with asymptomatic adult carriers surviving the disease that maintains the infectious pressure within the population [16,17].

Experimental challenge of Atlantic salmon (*Salmo salar* L.) with IPNV linked avirulent and virulent Sp serotype strains to specific amino acids in the VP2 protein represented by motifs P_217_T_221_ and T_217_A_221_, respectively [18]. However, the avirulent motif was associated with both clinical and subclinical infections of different fish species under field conditions [19,20,21,22,23,24,25].

In Iran, the prevalence of all genogroups of IPNV was demonstrated in trout farms for the period from 2011 to 2013, without analyzing the virulence of isolates [3]. Moreover, sequence analysis of an IPNV isolate in 2012 revealed that the outbreak at the time was associated with avirulent or low pathogenic isolate belonging to genogroup 5 with a P_217_T_221_ motif in VP2 [2]. Although the prevalence and clinical outbreaks of the virus continue, as yet, no study has been carried out on the phylogeny and virulence properties of IPNV in Iran. In this study, the virulence motifs of IPNV isolated from five trout farms with unexplained mortality events in Iran from 2015 to 2017 were characterized.

## 2. Results

### 2.1. Clinical Finding and Histopathology

From May 2015 to June 2017, the causative agent of five of fourteen outbreaks in Kurdistan, Mazandaran, East Azerbaijan, Kermanshah, and Hamedan provinces in Iran were diagnosed as IPNV (Table 1). The other nine outbreaks were caused by the prevalent rhabdoviruses: Three infectious hematopoietic necrosis virus (IHNV), and six viral hemorrhagic septicemia virus (VHSV) cases [4,5].

**Figure 1:**
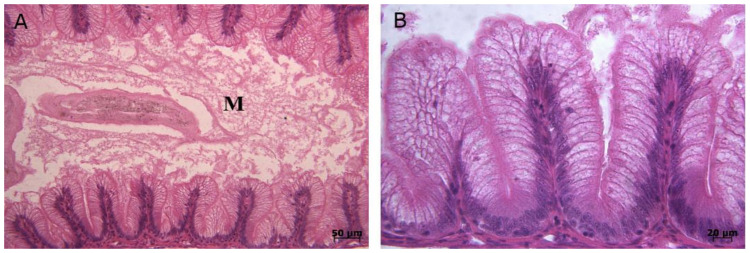
Histopathological lesions in tissue samples of trout infected with the IPNV. (**A**) Photomicrograph of the intestine with acute catarrhal enteritis. Notice blunting of villi. Excess mucous (M) is seen in the lumen. (**B**) High magnification of intestine with acute catarrhal enteritis.

Gross examination of infected fish were pale gills, bilateral exophthalmia, darkened skin, and mild abdominal distension. Internally, the intestine was void of food, but filled with yellow mucoid fluid, and the spleen was enlarged (Supplementary Figure S1). Microscopic examination of intestinal sections confirmed acute catarrhal enteritis and excess mucous secretion in all samples (Figure 1).

### 2.2. Viral Isolation and RT-PCR Assay

**Figure 2:**
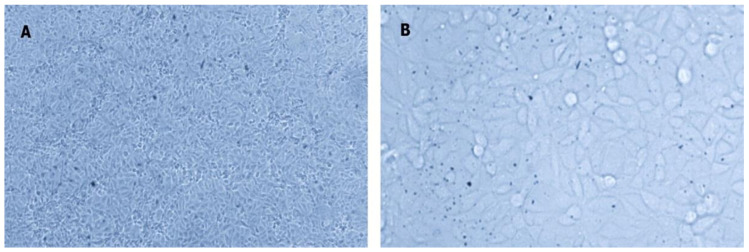
The cytopathic effect is caused by IPNV in the CHSE-214 cell line. (**A**) Control uninfected cells; (**B**) infected cells showing the typical cytopathic effect (CPE) for IPNV (spindle-shaped cells) at 7 dpi (40×).

The fish samples showed no parasites, and pathogenic bacteria were not isolated. However, tissue filtrates from the kidney of diseased trout produced the typical cytopathic effect (CPE) for IPNV (spindle-shaped cells) in the Chinook Salmon Embryo-214 (CHSE-214) cell culture (Figure 2). RT-PCR screening of tissue samples and CPE positive cell culture supernatants consistently produced the expected 405 bp fragments of IPNV-VP2, and the virus genome was confirmed by partial sequencing, and the GenBank BLAST search. The partial VP2 sequences were deposited in the NCBI GenBank (Table 1). Neither VHSV nor IHNV were detected in the IPNV positive samples.

### 2.3. Sequences Analysis

The partial VP2 sequences of the IPNV isolates showed high nucleotide and amino acid identity (≥96.7%) among themselves, and with other Iranian IPNV isolates (GU338037, KF279643, and KC489465) reported from 2009 to 2012. Isolate S.AV-IR-IPNV2 (KX665158) from the East Azerbaijan province showed the highest sequence divergence (3.3%).

The sequences of the Iranian isolates displayed the highest sequence identities (up to 100%) with sequences of Italian isolates (MG543599, MG543630, and MG543625), as well as above 98% similarity with sequences of isolates from Turkey (KY606210, KY986958, KY986960, and KY986964), Spain (AJ489222), Scotland (FN257531), and Ireland (KJ801314), all of which originated from farmed rainbow trout in freshwater, except for KY986964 that was from farmed common carp.

**Figure 3:**
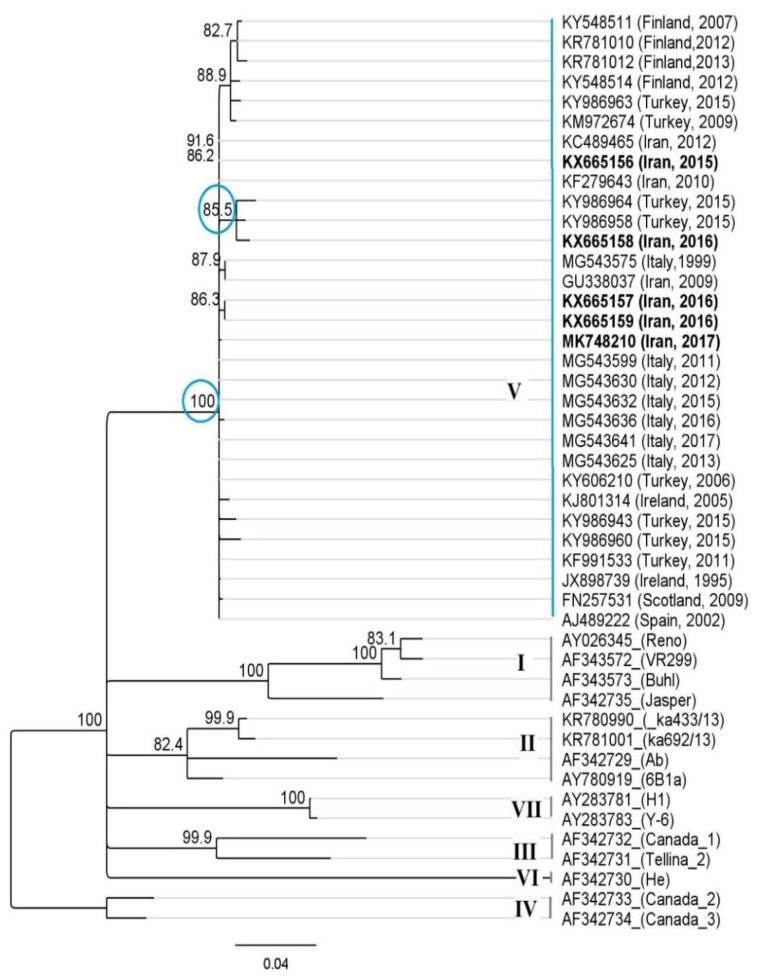
Phylogenetic analysis of Iranian isolate of IPNV detected in farmed trout (*O.mykiss*) based on the partial nucleotide sequences of the VP2 gene (405 bp). The phylogenetic trees were constructed using the Geneious Prime (neighbor-joining with the Hasegawa–Kishino–Yano (HKY) model of nucleotide substitution). The Iranian isolates of IPNV detected in this study (Acc No. KX665156-9 and MK748210) were classified as genogroup 5, serotype Sp (A2) with close identity to Turkish, European, and other Iranian isolates.

Phylogenetic analysis using IPNV-VP2 sequences derived from outbreaks in Iranian trout farms, as well as representative sequences from all of seven genogroups (41 isolates), revealed that the detected isolates belong to genogroup 5, and closely related to Sp serotype strains (European origin), as seen in Figure 3.

**Figure 4:**
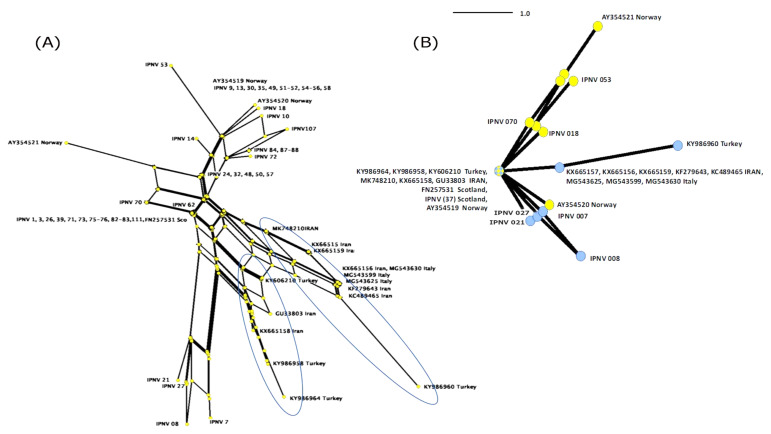
The neighbor network (**A**) and its derived Parsimony network (**B**) of non-homologous amino acid of VP2 of 15 IPNV sequences are listed in Table 2, and 57 IPNV sequences are analyzed in Ulrich et al. [26]. The highlighted branches indicate two clades of IPNV isolates in Turkey and Iran (**A**): Isolates IPNV 01-111 from Scotland [26], (**B**): IPNV (37) indicates 37 IPNV isolates. Yellow nodes: IPNV isolates of Atlantic salmon, Blue nodes: IPNV isolates of rainbow trout.

Isolate S.AV-IR-IPNV2 (KX665158) clustered close to two Turkish IPNV isolates (KY986958 and KY986964) with high bootstrap support. A haplotype analysis based on non-homologous amino acids of VP2 revealed that there indeed appear to be two lineages of IPNV present in the two neighboring main trout producing countries of the Middle East (Figure 4).

IPNV strains displaying T_217_A_221_ and P_217_A_221,_ in VP2 have been shown to be of high and moderate virulence, respectively; whereas, P_217_T_221_ has been associated with a non-virulent or low virulent nature [18]. Nonetheless, all Iranian isolates had proline and threonine at amino acid residues 217 and 221, respectively. Iranian isolates showed an extended P_217_T_221_A_247_ motif except for isolate S.AV-IR-IPNV2, which presented a P_217_T_221_E_247_ motif. The isolates also had the I_199_D_252_D/H_257_T_281_N_282_G/R_286_V_288_ and I_199_N_252_N_257_T_281_N_282_R_286_V_288_ (KX665158) motifs (Table 2 and Supplementary Figure S2).

## 3. Discussion

From 2015 to 2017, unexplained mortalities occurred on 14 farms in several provinces with major trout production in Iran. Based on the clinical signs, pathology, virus isolation, and molecular investigations, IPNV was diagnosed on farms in Kurdistan, Mazandaran, East Azerbaijan, Hamedan, and Kermanshah provinces.

Clinical signs reported in previous studies were also observed in infected fish of this study, specifically exophthalmia, abdominal distension, and skin blackening [34,35].

The acute disease is usually associated with necrosis of the acinar tissue of the pancreas and marked catarrhal enteritis of the intestinal mucosa [28,36,37]. However, the acinar tissue may be less affected or even regenerated [38]. Our histopathological findings confirmed acute catarrhal enteritis in the intestine of all moribund fish samples. Bacterial, parasite, and other viral pathogens were excluded as the cause of lesions, due to the negative results of the examinations.

The recommended method for the diagnosis of IPNV is the isolation of the virus in cell culture, followed by the antibody or molecular identification, which is mainly carried out using head kidney samples [39]. Nonetheless, several studies have shown that PCR-positive samples from IPNV carriers may be negative in cell culture, due to a low viral load in the samples [21,40,41]. RT-PCR assays performed on RNA extracts of tissue filtrate of infected fish and viral culture samples in this study yielded consistent results, suggesting a high viral load in the tissues sampled from moribund trout.

The VP2 gene is often used in phylogenetic studies of the IPNV genome [13,18,19] and has been shown to carries the determinants of virulence motifs inside an immune dominant region [18,42,43].

Six genogroups (I-VI) of IPNV correlating with serotypes and geographic origins have been described based on the VP2 sequences [13]. A seventh genogroup (VII) comprising Japanese aquabirnaviruses has also been proposed based on the VP2/VP4 junction [14].

In Europe, most IPN outbreaks in salmonids have been associated with genogroup 5 (Serotype Sp) [13,19]. IPNV genogroup 5 has also shown to be more virulent in rainbow trout than genogroup I [35] and genogroup II [44].

The Iranian isolates are also belonging to genogroup 5 strains clustering within known serotype Sp strains with high identity to Turkish and European isolates. They also show a high similarity with other Iranian IPNV isolates from trout fry [2,30]. The recent study of Buyukekiz et al. [23] has also described the prevalence of genogroup 5 (Sp) isolates with the P_217_T_221_ motif on Turkish trout farms.

The S.AV-IR-IPNV2 (KX665158) isolate from the East Azerbaijan province near the western border of Turkey clustered with two isolates from Turkey (KY986958 and KY986964), while showing the highest sequence difference with other Iranian isolates. The Turkish annual freshwater trout production is similar in volume to that of Iran, and the aquaculture industry in Turkey also relies on importing eyed eggs from Europe [23].

Vertical transmission of IPNV can occur via the fertilized eggs of trout [34]. The phylogenetic and network analyses indicate that the imported eyed eggs to both Turkey and Iran have led to the inadvertent importation of IPNV from European sources. However, it may also suggest some cross border exchange of material between Turkey and Iran.

IPN mortality rates were inconsistent at different farms and ranged from 50–60% (Hamedan) to 20–30% (Kurdistan) in fish of 1 g and 10g, respectively, confirming the age dependence of mortality caused by IPNV [7,10,34].

IPNV is represented by various strains with different virulence associated with the hypervariable domain of the VP2 capsid. Strains show several conspicuous amino acid motifs located at positions 199, 217, 221, 247, 250, 252, 281, 282, 286, 288, 321, and 500, among which 217 and 221 have mainly been considered and experimentally shown to be associated with virulence status [18,27,28,29,33]. The VP2 of the IPNV isolates in this study all presented a P_217_T_221_ motif that previously has been associated with avirulence or low virulence in Atlantic salmon (Table 2), whereas all IPNV-positive and clinically affected fish showed moderate mortalities. This result is consistent with previous observations in farmed rainbow trout in freshwater [2,19,22,23,24,25].

Rainbow trout have shown significant genetic variation in resistance against IPNV with mortalities ranging from 0% to 100% [45]. Mutoloki et al. [29] have also reported that the extended motif P_217_T_221_A_247_ is associated with subclinical infection of *A. salmon* in seawater. Therefore, our evidence may be due to other intrinsic virological and/or host features.

Four of five IPNV isolated from farmed fish showing moderate mortality had an extended P_217_T_221_A_247_ motif, and only one isolate had the P_217_T_221_E_247_ motif (one nt switch: A > C) like that already reported for Turkish strains [23].

Since the P_217_T_221_E_247_ motif is located in the hypervariable and the immunodominant region of VP2, it suggests a possible case of emergence of virulence driven by host immune response [46].

In conclusion, our results indicate that the moderately pathogenic IPNV isolates belong to genogroup 5 of European origin with an extended P_217_T_221_ motif in VP2 were associated with mortality outbreaks in Iranian trout farms from 2015 to 2017. Moreover, the prevalence of highly homologous isolates of only one genogroup may indicate a frequent exchange of the virus between farms from limited sources. This suggests the necessity for improvement of biosecurity for trout aquaculture in Iran, especially for the movement of eggs and fish. Additional studies are needed to describe the pathogenicity of these IPNV isolates in more detail.

## 4. Materials and Methods

### 4.1. Fish and Laboratory Examination

Moribund rainbow trout (1–10 g) specimens were collected from 14 outbreaks with high mortality, which occurred in the provinces with major trout production in the east and north of Iran from 2015 to 2017. All the procedures were performed according to guidelines for the care and use of animals as given by the University of Tehran Ethics Committee for Animal Experimentation. Mortality rates were 20% to 60% at water temperatures of 12 °C to 15 °C. Wet mounts of the gills and skin were prepared for parasitological examinations. Bacterial culture from the kidney tissue was performed on tryptic soy agar (TSA) incubated at 24 °C for up to 72 h. Pools of kidney tissues from the maximum five fish were diluted 1:10 in transport medium (Eagle’s Minimum Essential Medium, pH 7.6, supplemented with 10% newborn bovine serum and 100 µg/mL gentamicin) and used for viral isolation and molecular confirmation. The gastrointestinal tract samples were also fixed in 10% neutral buffered formalin (NBF) for histopathological examinations.

### 4.2. Histopathology

For histological purposes, samples of intestine tissue preserved in the 10% NBF were dissected, dehydrated, and embedded in paraffin with a paraffin tissue processor and paraffin dispenser (Did Sabz Co.; Orumiyeh, Iran), sectioned at 5 µm and stained with hematoxylin-eosin (H&E). Sections were observed with light microscopy, and representative images were taken using a microscope camera (uEye; UI-2250; GmbH, Obersulm, Germany).

### 4.3. Virus Isolation

Pooled tissue samples of kidney in the transport medium were homogenized by sterile steel beads (5 mm) and using a TissueLyser (Qiagen, GmbH, Haan, Germany) for 5 min at 25 Hz, and then centrifuged (3000× *g* for 10 min). The supernatants were inoculated onto CHSE-214 cells (1:10 dilutions), cultured in Minimal Essential Medium (MEM) supplemented with 10% FBS, L-glutamine, 100 IU penicillin G, and 100 μg/mL of streptomycin at 25 °C. The inoculated cell cultures were incubated at 15 °C for two weeks and were examined daily for cytopathic effects. The supernatants of cultures developing positive CPE were sampled and processed for RT-PCR.

### 4.4. RT-PCR and Sequences Analysis

RNA was extracted from 20 mg of the homogenized pooled samples and from 200 µL of the IPNV positive CPE-supernatant samples using the RiboEx SL Total RNA extraction kit and Exgene^TM^ Viral DNA/RNA kit, respectively (GeneAll, Seoul, Korea). The cDNA synthesis (total volume 25 μL) was carried out from 5 μL of the extracted RNA using the HyperScript^TM^ First strand Synthesis Kit (GeneAll, Korea) according to the manufacturer’s recommendations. 

Primer pairs, SVP2-F (5′GTTCGACAAGCCATACGTCC 3′), and SVP2-R (5′GCTTGGTGATGTTCTCGGTC 3′) were designed and used for RT-PCR amplification of the variable region of VP2 (nt507–nt910) containing the virulence residues of 217, 221, and 247. PCR amplification was performed in a final volume of 25 μL containing 12.5 μL Taq DNA Polymerase Mix Red (GeneAll, Korea), 2 μL of cDNA, 1 μL of each primer pair (10 pmol), and 8.5 μL nuclease-free water. Amplification was carried out with 40 cycles of 94 °C for 30 s, 58 °C for 30 s, and 72 °C for 45 s followed by a final extension at 72 °C for 10 min. The amplification products were resolved by electrophoresis using a 1% agarose under UV light. All samples were also screened for IHNV, VHSV using RT-PCR assay, according to Ahmadivand et al. [5]. The amplified products of kidney tissue samples were purified using the *Expin*^TM^ *PCR SV kit* (Gene all, Korea), subjected to nucleotide sequence analysis by the dideoxy chain termination method (Applied Biosystems, Foster City, CA, USA). Nucleotide sequences were analyzed using Geneious Prime (http://www.geneious.com/) and BioEdit software [47], and the National Centre for Biotechnology Information (NCBI) BLAST tool. The neighbor-joining method and the HKY model of nucleotide substitution using 1000 bootstrap replicates were performed for phylogenetic tree construction. The neighbor net and Parsimony net analysis were done in SPLITS TREE 4.0 after deleting homologous amino acid columns from the alignment in MEGA7, leaving a character set of 19 amino acids [48].

## Figures and Tables

**Table 1:** Description of infectious pancreatic necrosis virus (IPNV) outbreaks in Iranian trout farms included in this study.

Isolate ID	Outbreak Date	Province	Weight (g)/T (°C) ^1^	Mortality (%) ^2^	Acc No.
S.AV-IR-IPNV	Jul 2015	Mazandaran	1/12	30–40%	KX665156
S.AV-IR-IPNV1	May 2016	Kermanshah	2/12	40–60%	KX665157
S.AV-IR-IPNV2	Jun 2016	East Azerbaijan	7/13	30–40%	KX665158
S.AV-IR-IPNV3	May 2016	Kurdistan	10/12	20–30%	KX665159
S.AV-IR-IPNV4	Nov 2017	Hamedan	1/11	50–60%	MK748210

^1^ Average weight/water temperature at the time of outbreak; ^2^ Cumulative mortality (based on farm owner information).

**Table 2:** VP2 amino acid patterns of Iranian IPNV isolates compared to foreign isolates and the virulent reference strains. The listed amino acid positions (except 257) have been proposed as virulence motifs of the Sp serotype [18,27,28,29].

Isolates	Genogroup/Serotype	VP2 Amino Acid Position													
		199	217	221	247	252	257 ^a^	281	282	286	288	Host	Year	Acc. No	References
S.AV-IR.IPNV	5/Sp	I	P	T	A	D	H	T	N	G	V	*O. mykiss*	2015	KX665156	This study
S.AV-IR.IPNV1	5/Sp	I	P	T	A	D	D	T	N	G	V	*O. mykiss*	2016	KX665157	This study
S.AV-IR.IPNV2	5/Sp	I	P	T	E	N	N	T	N	R	V	*O. mykiss*	2016	KX665158	This study
S.AV-IR.IPNV3	5/Sp	I	P	T	A	D	D	T	N	G	V	*O. mykiss*	2016	KX665159	This study
S.AV-IR.IPNV4	5/Sp	I	P	T	A	D	D	T	N	R	V	*O. mykiss*	2017	MK748210	This study
IRIPNV	5/Sp	I	P	T	A	D	H	T	N	G	V	*O. mykiss*	2012	KC489465	[2]
IRAN (SP)	5/Sp	I	P	T	A	D	H	T	N	G	V	*O. mykiss*	2010	KF279643	[30]
IRAN-2009	5/Sp	I	P	T	E	N	H	T	N	R	V	*O. mykiss*	2009	GU338037	[31]
Italian Isolates	5/Sp	I	P	T	A	D, N	H	T	N	G, R	V	*O. mykiss*	1999–2017	75 isolates	[25]
Turkish Isolates	5/Sp	I	P	T	A, E	D, N	H, D	T	N	G, R	V	*O. mykiss*	2006–2015	30 isolates	[23]
Finnish Isolates	5/Sp	I	P	T	A	D, N	D	T	N	R	V	*O. mykiss*	2007–2013	6 isolates	[22]
Sp (1164)	5/Sp	I	P	T	A	N	H	T	N	R	V	*O. mykiss*	2002	AJ489222	[32]
Low: (Sp103)	5/Sp	I	P ^b,c^	T ^b,c^	A ^b,c^	V, N ^c^	D	T, S ^c^	N, D ^b^	R	V	*S. salar*	2004	AY354519	[27]
Moderate: (Sp116)	5/Sp	I	P ^b,c^	A ^b,c^	A ^b,c^	V	D	T	N	R, A ^d^	A	*S. salar*	2004	AY354520	[27]
High: (Sp122)	5/Sp	T	T ^b,c^	A ^b,c^	T ^b,c^	V	D	T	N	R, K ^d^	V	*S. salar*	2004	AY354521	[27]

^a^ Variable position in Iranian isolates; ^b^ Santi et al. [18]; ^c^ Mutoloki et al. [29]; ^d^ Bruslind and Reno [33]. Additionally, the P_217_T_221_A_247_ motif was found in 4 IPNV isolates of *O. mykiss* and in 20 isolates from *S. salar* from Scotland. All these isolates were in genogroup 5 [26].

## Supplementary Materials

The following are available online at https://www.mdpi.com/2076-0817/9/10/782/s1, Figure S1: Gross lesions in the infected rainbow trout with infectious pancreatic necrosis virus; Figure S2: Multiple sequence alignment Iranian isolates of IPNV detected in farmed trout (*O.mykiss*) based on the partial (405bp) nucleotide sequences of VP2 gene.
