# Supplementary material for: Low Pathogenic Strain of Infectious Pancreatic Necrosis Virus (IPNV) Associated with Recent Outbreaks in Iranian Trout Farms
Source: Pathogens. 2020 Sep 24;9(10):782. doi: 10.3390/pathogens9100782 (PMC7650613; doi:10.3390/pathogens9100782)
Supplement: Supplementary file 1 [file pathogens-09-00782-s001.zip › Supplementary Data -IPNV MS.docx]

**
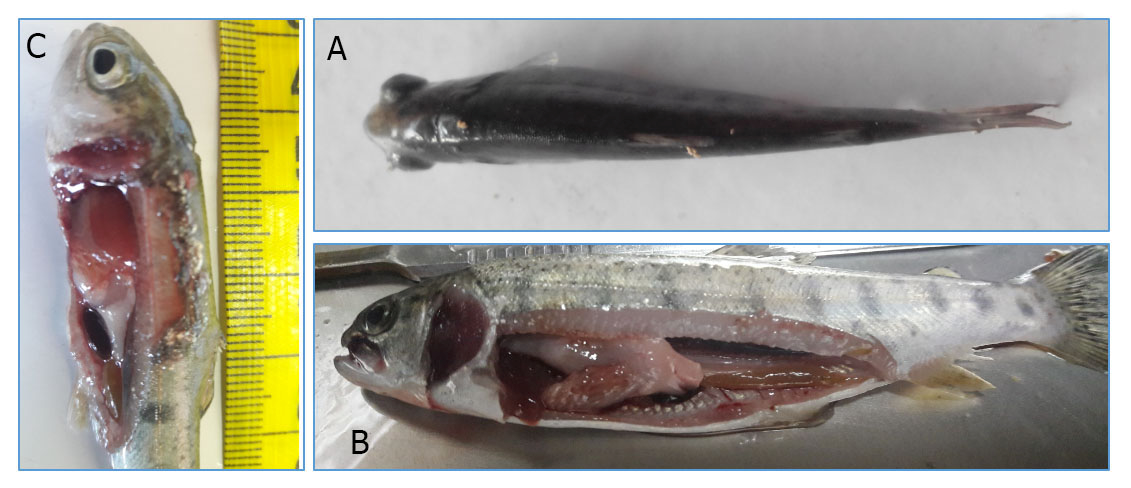
**

**Supplementary Fig.1.** Gross lesions in the infected rainbow trout with infectious pancreatic necrosis virus. Lesions included darkening of the skin and exophthalmia (A); Yellowish fluid in the intestine (B & C). Pale gills, and enlarged spleen (C).

**
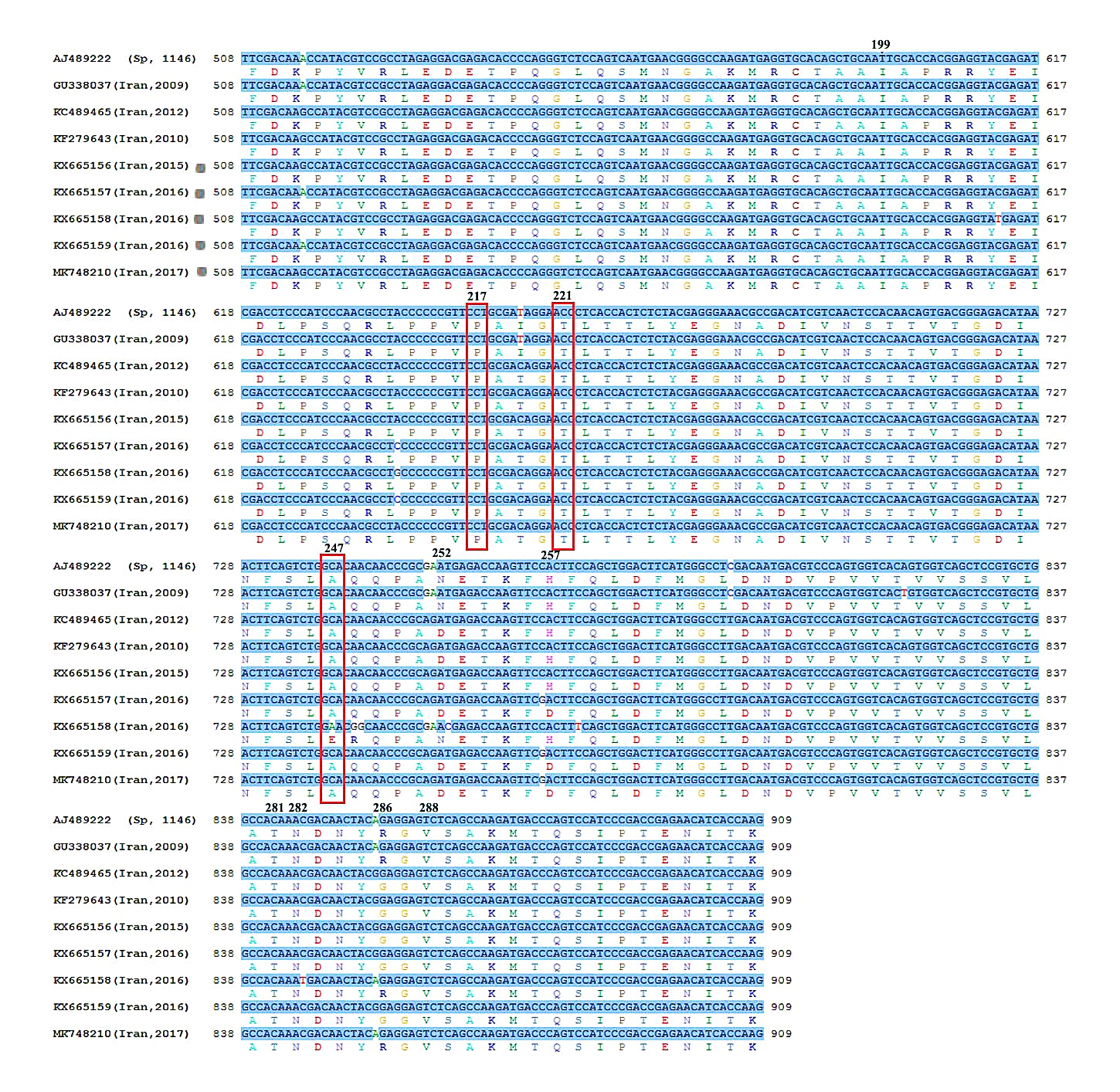
 Supplementary Fig.2.** Multiple sequence alignment Iranian isolates of IPNV detected in farmed trout (*O.mykiss*) based on the partial (405bp) nucleotide sequences of VP2 gene. Nucleotide sequences were aligned using the Geneious Prime and BioEdit Software. The residues at positions 217, 221, and 247 in VP2 involved in IPNV virulence (Red rectangular). The Iranian isolate of IPNV detected in this study (Acc No. KX665156-9 and MK748210; Marked by ■) showed P_217_T_221_A_247_ and P_217_T_221_E_247_ (KX665158) motifs.
